# Non-native red alga *Gracilaria vermiculophylla* compensates for seagrass loss as blue crab nursery habitat in the emerging Chesapeake Bay ecosystem

**DOI:** 10.1371/journal.pone.0267880

**Published:** 2022-05-31

**Authors:** Megan A. Wood, Romuald N. Lipcius

**Affiliations:** Virginia Institute of Marine Science, William & Mary, Gloucester Point, Virginia, United States of America; University of Sydney, AUSTRALIA

## Abstract

Non-native species can become deleterious or potentially beneficial as components of novel ecosystems. The non-native red macroalga *Gracilaria vermiculophylla* may provide nursery habitat where eelgrass *Zostera marina* has been extirpated in Chesapeake Bay. A mensurative experiment was conducted monthly May–October 2013 and 2014 in the York River, Chesapeake Bay, to evaluate hypotheses that *Gracilaria* (1) can compensate for the loss of seagrass nurseries by colonizing habitats where seagrass has been eliminated by environmental stress, and (2) is utilized by juvenile blue crabs (*Callinectes sapidus*) as nursery habitat. We quantified *Gracilaria* presence, percent cover, and biomass as a function of *region* (upriver, midriver, and downriver) and *seagrass presence or absence* using stratified random sampling, 20-m transects, and 0.0625-m^2^ quadrats. *Gracilaria* volume was measured and converted to dry weight. Effects of the factors and covariates temperature, salinity, dissolved oxygen, month, and year were analyzed using generalized linear models. Juvenile blue crab density was quantified in summer 2013 using suction sampling in *Gracilaria* and seagrass. A model with the collective effect of *region* and *seagrass presence or absence* (downriver seagrass, downriver unvegetated bottom, midriver unvegetated bottom) best predicted *Gracilaria* abundance. *Gracilaria* presence, percent cover, and biomass were highest in downriver seagrass, followed by downriver unvegetated bottom, and then midriver unvegetated bottom, where seagrass has been extirpated, supporting hypothesis (1). *Gracilaria* did not occur upriver, likely due to a lack of recruitment. Seagrass and *Gracilaria* housed similar densities of juvenile blue crabs, supporting hypothesis (2). We estimated that a single 40-ha cove system with *Gracilaria* could house 200,000 juvenile crabs as would a single 2.4-ha seagrass bed. Consequently, the numerous midriver and downriver cove systems in the York River could support millions of young juvenile blue crabs and thereby compensate for the loss of seagrass in the river and in other areas of Chesapeake Bay. At present, *Gracilaria* has no widespread negative impacts on seagrass in the York River or most regions of Chesapeake Bay, likely because percent cover and biomass are not excessively high at present. We posit that *Gracilaria* has become an important alternative nursery habitat for the blue crab in Chesapeake Bay and can potentially mitigate impacts of climate change on seagrass nursery habitats.

## Introduction

Coastal and estuarine systems are often the most degraded systems worldwide due to increased human activity along coastlines [1, 2], which renders these systems susceptible to colonization by non-native species [3], which are often harmful to the ecosystems they colonize, both ecologically and economically [4–6]. However, non-native species may benefit degraded systems by restoring lost functions [7]. For instance, the green crab *Carcinus maenas* has facilitated salt marsh recovery in some areas where it is not native by reducing consumption of cordgrass by native species [8]. Introduced plants can increase structural heterogeneity and provide novel habitat that can profit native species [9]. For example, the green alga *Codium fragile* sp. *tomentosoides* increased recruitment of native Mediterranean mussels (*Mytilus galloprovincialis*) in the Adriatic Sea [10]. In previously unvegetated intertidal flats along the southeastern Atlantic Coast of North America, the red macroalga *Gracilaria vermiculophylla* (synonym *Agarophyton vermiculophyllum*) attracted epifaunal colonizers by adding structure when facilitated by the polychaete *Diopatra cuprea* [11–13], increased epifaunal abundance and richness [14], and provided similar detrital habitat as native smooth cordgrass *Spartina alterniflora* wrack for invertebrates [15]. In Europe, *G. vermiculophylla* increased epifaunal species richness when entangled in seagrass beds (eelgrass *Zostera marina*) in Denmark [16] and increased macrofaunal richness and both macrofaunal and meiofaunal density in intertidal mudflats in France [17]. Thus, introduced macroalgae can enhance both unvegetated and native seagrass habitats.

Globally, seagrasses are in decline [18], and the fauna that use seagrasses as nursery habitats are thus threatened [19]. While seagrass habitats are susceptible to disturbances, both natural and anthropogenic, eutrophication is one of the primary causes of seagrass decline, which has led to macroalgal blooms worldwide [20, 21]. When seagrass beds deteriorate, the resultant unvegetated substrate can then be colonized by macroalgae [22], which may fill some of the ecological roles of seagrasses [23].

In lower Chesapeake Bay, eelgrass *Zostera marina* is the dominant seagrass on shallow shoals, and, along with widgeon grass *Ruppia maritima*, provides resources and protection to early life history stages of many animals including the blue crab *Callinectes sapidus* [24–28]. However, seagrasses have been in decline in Chesapeake Bay since the 1930s; historical declines were due largely to disease and storms [18, 29], while decreased water quality caused by anthropogenic nutrient and sediment inputs is the primary driver of more recent declines [30]. Increased fragmentation and decreased areal cover of seagrass beds may significantly lower recruitment of blue crab and other species that use seagrass as primary nursery habitat [26, 31, 32].

Similar to what has occurred in marine ecosystems worldwide [23], macroalgae may provide nursery habitat for blue crabs [33], other invertebrates, and fish, and thereby compensate for the loss of seagrasses. *Gracilaria vermiculophylla* (herein *Gracilaria*) is a non-native, coarsely branching, red macroalga originating from the Western Pacific [34] and which has colonized shallow coastal areas of the Atlantic Ocean along North America and Europe [12, 35–39]. Species within the family Gracilariaceae are often morphologically similar and difficult to differentiate [16, 40, 41]. In Chesapeake Bay and the seaside lagoons of Virginia and Maryland, the initial introduction and subsequent spread of *Gracilaria* was overlooked due to its cryptic morphological characteristics [18, 37, 42]. Although *Gracilaria* was first identified in Chesapeake Bay in 1998 via genetic barcoding [36], it is likely that the alga was introduced much earlier with the non-native oyster *Crassostrea gigas* as a source vector [43]. The alga has become ubiquitous in shallow areas and coves in the tributaries of Chesapeake Bay and seaside lagoons [20, 21, 37] and ranges along the east coast of North America from Georgia [12] to New Hampshire [44].

*Gracilaria* may act as a nursery habitat by providing both refuge from predation and increased food resources. Survival of juvenile blue crabs is enhanced in *Gracilaria* compared to both seagrass and unvegetated substrate [33]. Unattached algae also modify soft-bottom habitat [12, 16, 45–47] and can change the structure of associated communities by altering the physical, chemical, and biological processes within those habitats. At intermediate levels of algal biomass, this coarsely branching macroalga creates structural heterogeneity in colonized soft-bottom habitats, and may also provide new habitats and food resources for other organisms [12, 16, 38]. Thus, local species diversity may be enhanced by *Gracilaria* [48]. Drifting, unattached *Gracilaria* can also become entangled in seagrass beds, creating a mixed habitat that supports a higher diversity and abundance of invertebrate fauna by increasing heterogeneity or by improving habitat quality [16].

At high biomass, *Gracilaria* may be detrimental to both seagrasses and other organisms by forming dense mats, which can decrease light availability for seagrasses and cause hypoxia or anoxia [37, 49, 50]. Mats of *Gracilaria* may smother and kill the seagrass in which they are entangled, leaving the alga without protection from tidal currents and waves that remove it from the area, and thus cause a “habitat cascade” that is detrimental to fauna associated with both seagrass and *Gracilaria* [16]. Additionally, “superblooms” of *Gracilaria* have been associated with *Diopatra cuprea* declines on intertidal mudflats [51]. While dense mats are common in the seaside lagoons adjoining lower Chesapeake Bay [37], high densities of *Gracilaria* are generally limited to areas with low water flow in tributaries within Chesapeake Bay [33]. There is little evidence that *Gracilaria* is detrimental in Chesapeake Bay; therefore, it may be acting as a nursery habitat in this non-native area.

Nursery habitats like seagrass beds, macroalgae, and marshes produce chemical signals that cue megalopae (blue crab postlarvae) to the location of nursery habitats in lower Chesapeake Bay [52, 53]. Megalopae ride nocturnal flood tide currents upstream towards nursery habitats and rest near the bottom during ebb tides and daytime [54–56]. When megalopae reach nursery habitats, they metamorphose into the first benthic instar (J1) [57, 58]. Metamorphosis from the megalopal stage to J1 (about 3 mm carapace width, CW) and settlement are accelerated by cues from structured nursery habitats or lower salinities [53, 57, 59]. Juveniles typically remain in these habitats until they reach about 20–30 mm CW, after which they emigrate to unvegetated secondary nursery habitats like shallow mud coves [60, 61]. Structured habitats provide refuge from predation as well as abundant prey resources for early juvenile crabs [27, 28, 61, 62]. Emigration from structured habitats may be due to a lack of suitable refuges or food for larger juveniles [27, 28, 33, 61], or it may be density dependent [60, 63].

If *Gracilaria* is present in areas from which seagrasses have been extirpated due to environmental change or where juvenile blue crab recruitment is higher than seagrasses can support, it may represent an alternative, emerging primary or secondary nursery habitat in a novel ecosystem. Unfortunately, no data exist on the availability of *Gracilaria* and associated blue crab densities in shallow habitats of lower Chesapeake Bay to provide field evidence for the proposition above and to complement a small-scale manipulative experiment [33]. Thus, the objectives of this study were to determine (1) if *Gracilaria* is present in shallow habitats when juvenile crabs are recruiting and where structured habitat is now absent, (2) how *Gracilaria* abundance varies spatially or as a function of seagrass presence, and (3) if blue crabs are using *Gracilaria* as a nursery habitat, by quantifying the distribution and abundance of *Gracilaria* and juvenile blue crabs in a mensurative experiment in the York River, a tributary of lower Chesapeake Bay. We hypothesized that *Gracilaria* (1) could compensate for the loss of seagrass nurseries in areas where seagrasses have declined or may be lost in the future due to environmental stress, and (2) would be utilized by juvenile blue crabs as a structured nursery habitat.

## Materials and methods

### Study area

**Fig 1:**
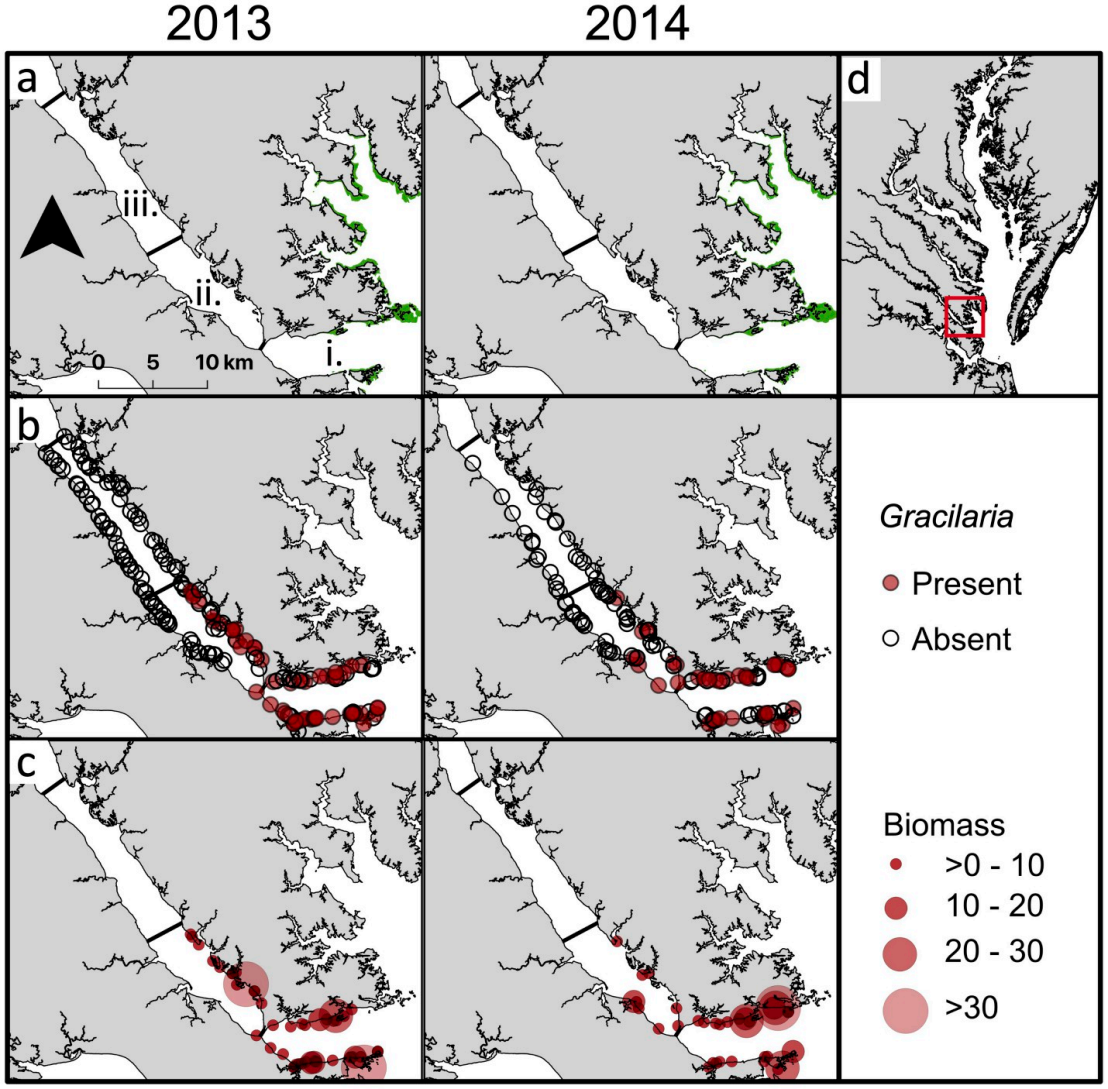
Map of York River (a) seagrass beds (green) in 2013 (left) [65] and 2014 (right) [66] and sampling regions at downriver (i), midriver (ii), and upriver (iii) sections along the river axis; (b) locations of all sites where *Gracilaria vermiculophylla* was present (filled) or absent (open) in 2013 (left) and 2014 (right); (c)*G. vermiculophylla* biomass (g dry weight m^−2^) at locations where it was present in 2013 (left) and 2014 (right); and (d) location of York River, a western shore tributary of Chesapeake Bay.

Studies were conducted in the York River in summer and early fall, 2013 and 2014. The study sites extended from the mouth of the York River where it meets Chesapeake Bay to 42 km upriver. The river was stratified along its salinity gradient into downriver, midriver, and upriver regions (Fig 1a). Seagrass is currently present downriver, it was present historically midriver but has since disappeared, and it does not occur nor has it occurred historically upriver [64].

Environmental data including salinity, dissolved oxygen, and water temperature were recorded once at each site using a YSI (Model 85, Yellow Spring Instruments), except for salinity at 6 sites (2 upriver, 4 downriver) in March 2014. For these 6 sites, salinity was estimated from the linear relationship between observed salinity and latitude and longitude during the sampling period (*y* = 3820.1 + 33.9(*latitude*) − 32.6(*longitude*); *r*^2^ = 0.93). Monthly mean salinity ranged from 19.6–20.6 downriver, 17.9–18.3 midriver, and 14.4–15.3 upriver. The values for salinity are similar to those derived from a long-term monitoring program over 5 y; Downriver: 21.2 ± 0.4 SE; Midriver: 18.8 ± 0.9; Upriver: 14.7 ± 0.9 (http://data.chesapeakebay.net/api.CSV/WaterQuality/WaterQuality/12–8-2016/12-8-2021/0,1/6/23/HUC12/1456,1459,1460/83), where SE = Standard Error of the Mean. Monthly mean water temperature ranged from 22.8–24.0°C downriver, 23.0–24.5°C midriver, and 24.9–25.0°C upriver. Hypoxic conditions (dissolved oxygen <2 mg L^−1^) were not observed at any sampling sites over the study period (Table 1), in concordance with historical data for these shallow habitats [67], and in contrast to deeper York River habitats where hypoxia occurs seasonally [68]. Large, continuous seagrass beds occur downriver and are dominated by eelgrass *Zostera marina* with widgeon grass *Ruppia maritima* scattered throughout and unvegetated substrate (mostly sand with some mud); both midriver and upriver are dominated by unvegetated substrate, although seagrass beds were common midriver until 1972 [29]. Depths were estimated using bathymetric data [69, 70]; all sites were located at <1.5 m depth below MLLW and ranged from the low intertidal to shallow subtidal.

**Table 1:** Summary of environmental data collected at sampled sites, including means of each variable during each sampling year; 95% confidence intervals are in parentheses.

	Downriver		Midriver		Upriver	
	2013	2014	2013	2014	2013	2014
**Temperature (°C)**	24.7	24.0	24.6	24.5	25.2	24.9
	(23.8, 25.5)	(22.9, 25.2)	(23.7, 25.4)	(23.5, 25.5)	(24.5, 25.9)	(23.3, 26.4)
**Salinity**	19.4	20.6	17.6	17.8	14.4	14.7
	(19.0, 19.9)	(20.1, 21.1)	(17.2, 18.1)	(16.9, 18.8)	(13.7, 15.0)	(12.9, 16.4)
**Dissolved Oxygen (mg/L)**	8.1	8.0	7.0	7.8	7.0	7.5
	(7.4, 8.9)	(7.4, 8.5)	(6.7, 7.4)	(7.5, 8.1)	(6.5, 7.5)	(7.1, 7.9)

### Mensurative experiment

The distribution and biomass of *Gracilaria* were quantified in the York River over two years, from May to October in 2013 and 2014. Sites were selected using a stratified random sampling design limited to shallow water areas (<1.5 m depth MLLW) with the three regions serving as strata. In 2013, 10 sites were selected in each region each month. In 2014, each month 7–8 sites were selected downriver and midriver, while only 4 sites were selected upriver due to its absence there in 2013. In the downriver region, sites included those with and without seagrass present.

At each site, three 20-m transects were set parallel to shore spaced at an interval of approximately 3 m relative to the shoreline. Transect depth ranged from approximately 0–2 m below MLLW. Transects were marked every meter, at which the vegetation present was noted. Five haphazard quadrats (0.0625 m^2^) were set along each transect. Within each quadrat, the percent cover of any vegetation was recorded, and, if it was present, *Gracilaria* was removed from the quadrat and its volume measured in a graduated cylinder to the nearest mL. *Gracilaria* volume was converted to biomass (g dry weight, DW) using a linear regression (*y* = 0.138*x*; *r*^2^ = 0.998) (S1 Fig).

### Juvenile crab density and size

**Fig 2:**
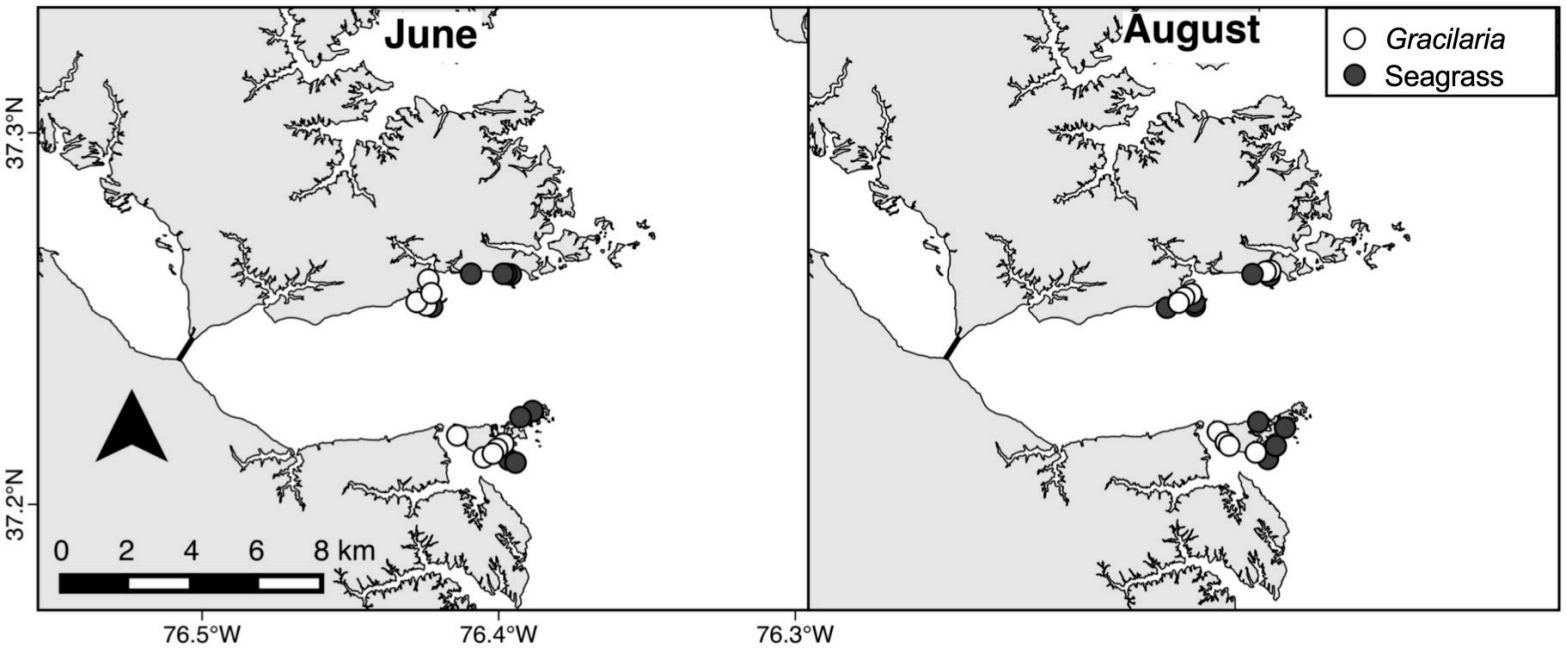
Map of crab sampling locations in June and August in the downriver area of the York River.

Juvenile blue crab (*Callinectes sapidus*) density in seagrass and *Gracilaria* was assessed in June and August 2013 in the downriver region (Fig 2). Sampling sites were haphazardly selected in shallow subtidal locations at depths between 0.1 and 1.1 m MLLW. During each sampling event, juvenile blue crabs were collected (n = 9–10 in each habitat) using the standard drop-net suction method [24]. Drop nets (1.68 *m*^2^) were suctioned for 6 min each, and crab densities were corrected for 78% efficiency. Seagrass beds were comprised predominantly of *Zostera marina* with some *Ruppia maritima*. Seagrass and *Gracilaria* samples both ranged from 30–100% cover.

Juvenile blue crabs were removed from all samples, counted, and measured to the nearest 0.1 mm using Vernier calipers. Crab densities were calculated for crabs <30 mm CW. Crabs were then dried at 60°C for at least 48 h before being combusted at 550°C for 5 h to determine ash-free dry weight (AFDW).

### Analysis

#### *Gracilaria* presence, percent cover, and volume

Nine logistic regression models (g_1_–g_9_) were developed to predict *Gracilaria* presence (Table 2), recorded as either 1 (present) or 0 (absent) at each site, as a function of base habitat (3 levels: downriver seagrass, downriver sand, midriver sand), month (6 levels: May–October) and year (2 levels: 2013 and 2014). The upriver treatment was eliminated from the analysis because *Gracilaria* never occurred upriver, as was the midriver seagrass treatment because seagrass did not occur midriver. Each model produced a log-likelihood value, which was then used to calculate Akaike’s Information Criterion (AIC) [71]. AIC_*c*_ values were used to correct for bias due to low sample size [71]. From these, Δ_*i*_ values and model probabilities (w_*i*_) were generated to compare the fit of the candidate models (g_*i*_) with the model having the lowest AIC_*c*_. A model was eliminated when w_*i*_ < 0.10 [71]; the individual parameter estimates of the best model were then evaluated. The best model was tested against other models, including the null and global models, with a Likelihood-Ratio *X*^2^ test (LR test).

**Table 2:** Information theoretic analysis [71] of 9 logistic regression models (g_*i*_) using base habitat (H), water depth (D), month (M), and year (Y) as predictors of *Gracilaria vermiculophylla* presence, where k is the number of parameters in a model, AIC is the Akaike information criterion, AIC_*c*_ is the corrected AIC, Δ_*i*_ is the difference between any model and the best model in the set, and w_*i*_ is the model probability. Models g_8_ and g_9_ are the global model and null model, respectively.

Model	k	AIC	AIC_*c*_	Δ_*i*_	w_*i*_
g_1_: Y + H	4	265.7	265.9	1.09	0.33
g_2_: M + H	8	269.2	269.9	5.04	<0.05
g_3_: Y + M + H	9	270.07	271.0	6.13	<0.05
g_4_: Y + M + H + D	10	271.8	272.9	8.06	<0.05
g_5_: Y*M + Y*H + M*H + D	27	274.5	282.7	17.88	<0.05
g_6_: Y*M + Y*H + D	17	271.3	274.4	9.59	<0.05
g_7_: H	3	264.7	264.8	0.00	0.58
g_8_: Y*M*H + D	37	287.7	303.8	39.0	<0.05
g_9_: mean	1	287.5	287.5	22.71	<0.05

The same set of models was used for *Gracilaria* percent cover and biomass, and the best model for these was the same as that for *Gracilaria* presence. Consequently, we only present the final model’s parameter estimates for *Gracilaria* percent cover and biomass. We also evaluated changes in percent cover and biomass by the magnitudes and confidence intervals of sample means over time and region. For these variables, we were primarily interested in whether or not *Gracilaria* would occur in the midriver and downriver regions during the main settlement and recruitment period from July through September, and to generate estimates of juvenile crab abundance using a combination of *Gracilaria* percent cover and the sampled crab densities quantified below.

#### Juvenile crab density and size

Juvenile blue crab densities for crabs <30 mm CW were analyzed using a Generalized Linear Model (GLM) with a log link and gamma family, due to the heavy right-skewed distribution of crab density. Twelve models (g_1_–g_12_) were developed to predict crab density as a function of habitat % cover, plant biomass, water depth, month and habitat type (seagrass or *Gracilaria*) (Table 3), and analyzed as described above. Plant % cover and plant biomass were not correlated (Pearson correlation test, *p* >>0.5) and could thus be included together in models.

**Table 3:** Information theoretic analysis [71] of 12 GLM models (g_*i*_) using habitat type (H), month (M), water depth (D), plant % cover (C) and plant biomass (B) as predictors of juvenile blue crab density, where k is the number of parameters in a model, AIC is the Akaike information criterion, AIC_*c*_ is the corrected AIC, Δ_*i*_ is the difference between any model and the best model in the set, and w_*i*_ is the model probability. Model g_9_ is the global model and model g_10_ is the null model.

Model	k	AIC	AIC_*c*_	Δ_*i*_	w_*i*_
g_1_: H + M	4	225.5	226.8	2.53	0.10
g_2_: H + M + C	5	222.3	224.2	0.00	0.34
g_3_: H + M + D	5	227.5	229.5	5.20	<0.01
g_4_: H + M + C + D	6	224.3	227.1	2.80	0.08
g_5_: H + M + C + B	6	221.9	224.7	0.45	0.27
g_6_: H + M + C + B + D	7	223.9	227.7	3.48	<0.01
g_7_: H*M + C	6	224.1	226.9	2.65	0.09
g_8_: H*M + C + D + B	8	225.6	230.7	6.45	<0.01
g_9_: H*C + M*C + D	8	227.2	232.3	8.09	<0.01
g_10_: H*B + M*B + D	8	228.0	233.2	8.93	<0.01
g_11_: H*C + M*C + H*B + M*B + H*D + M*D + D	14	233.1	252.2	27.95	<0.01
g_12_: mean	2	247.1	247.5	23.21	<0.01

## Results

### *Gracilaria* and seagrass distribution

**Fig 3:**
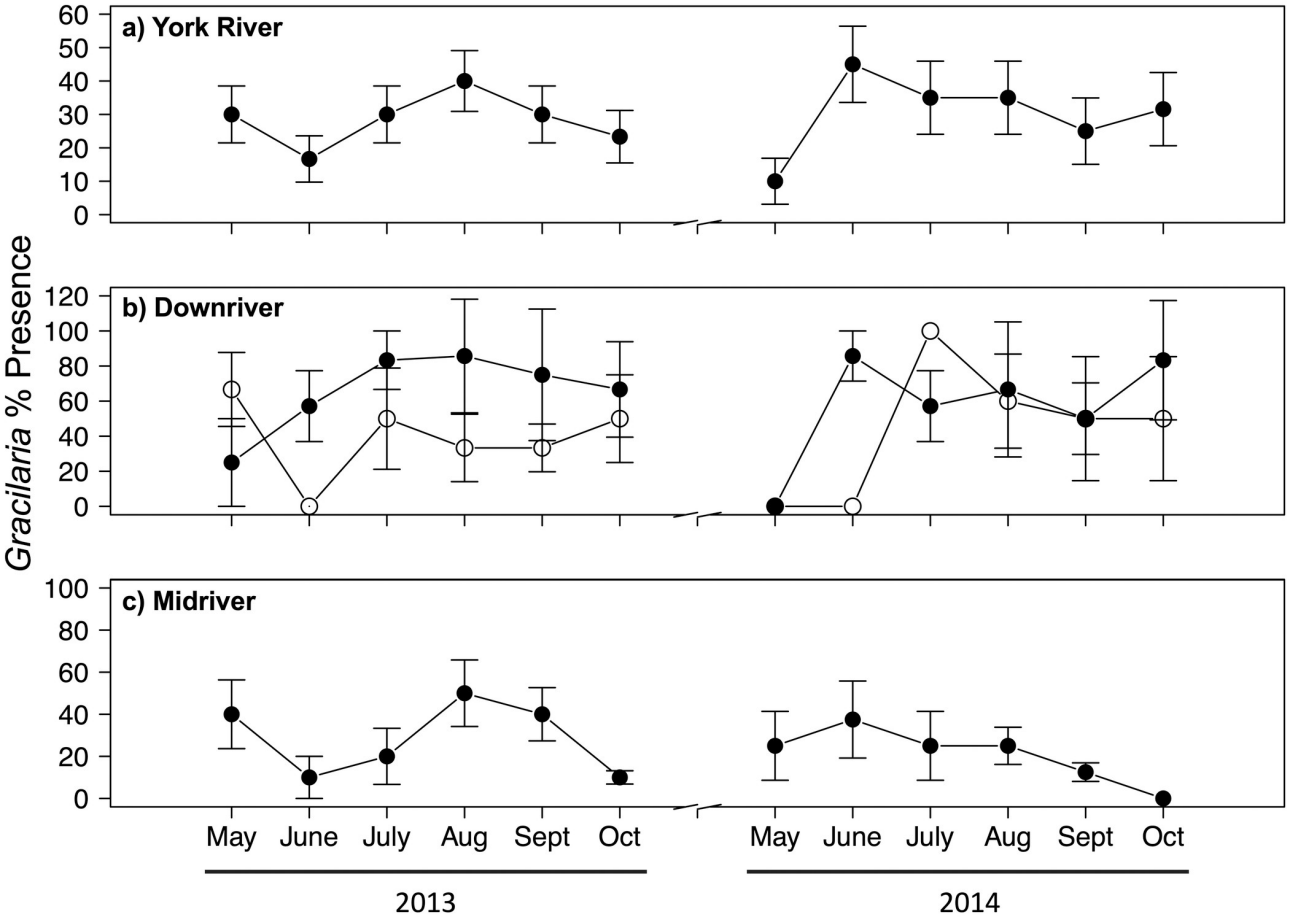
Mean percent presence of *Gracilaria vermiculophylla* during 2013 and 2014 over: (a) the entire York River;(b) the downriver region, where base habitats are seagrass (filled) and unvegetated (open); and (c) midriver region. *Gracilaria* was not observed upriver. Vertical bars represent 1 standard error of the mean. Note differing scales on y-axes.

In both years, *Gracilaria* was present at more sites downriver than midriver and never present upriver (Fig 1b). In the midriver zone, it was present primarily along the north shore of the York River (Fig 1b) where it occurred as unattached drift algae, incorporated into worm tubes, and less commonly, attached via holdfasts to shell fragments. Similarly, *Gracilaria* biomass was highest downriver and midriver along the north shore (Fig 1c). In downriver samples, *Gracilaria* typically occurred as unattached mats and less commonly integrated into worm tubes or attached to shell fragments. In contrast, seagrass only occurred in the downriver zone (Fig 1a). *Gracilaria* was present on average at 30.8% of sites in 2013 and at 30.3% of sites in 2014 (Fig 3). Algal presence was greatest overall in June 2014 at 45% of sites, downriver in June and October 2014 (75%), and midriver in August 2013 (50%).

Of the 9 candidate models predicting presence (Table 2), model g_7_ had the highest w_*i*_, although g_1_ deserved consideration because its w_*i*_ value exceeded 0.1 (Table 2). Model g_7_ only included base habitat, and the model fit was not improved by adding year in model g_1_ (LR test, *p* = 0.32). The global model g_8_ did not improve the fit (LR test, *p* = 0.10), whereas model g_7_ improved the fit significantly over the null model g_9_ (LR test, *p* ≪ 0.001).

**Fig 4:**
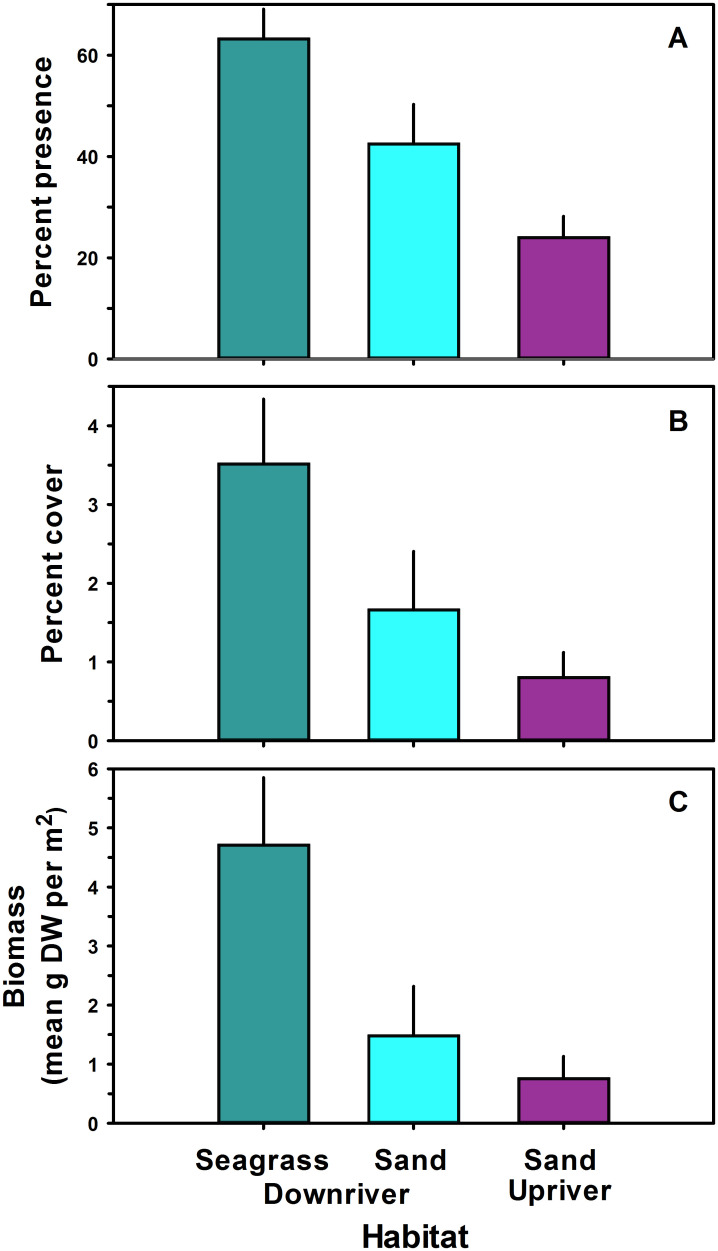
Mean percent presence (A), percent cover (B) and biomass (C) of *Gracilaria vermiculophylla* in downriver seagrass, downriver sand and midriver sand sites. Vertical bars represent 1 standard error of the mean.

*Gracilaria* presence in the York River was influenced by region and whether seagrass was present (Table 4). *Gracilaria* presence (63.2%) was 2.3 × higher in downriver seagrass than in downriver sand (42.5%) and 5.4 × higher than in midriver sand (24.0%) (Fig 4A). *Gracilaria* presence in downriver sand was 2.3 × higher than that in midriver sand (Fig 4A). While there was interannual variability in *Gracilaria* presence, with greater algal presence in 2013 than 2014 (Fig 3), it also varied by month but was present during the major blue crab recruitment period from July through September.

**Table 4:** Summary of GLM for model g_7_ predicting presence, including parameter estimates (log-transformed), standard errors, *z* values and *p* values. The intercept represents downriver seagrass. Model *g*_7_ explained 9.3% of the null deviance.

Coefficients	Estimate	SE	*z*	*p*
Intercept	0.54	0.25	2.16	0.03
Downriver sand	-0.84	0.41	-2.08	0.04
Midriver sand	-1.69	0.34	-4.97	≪ 0.01

**Fig 5:**
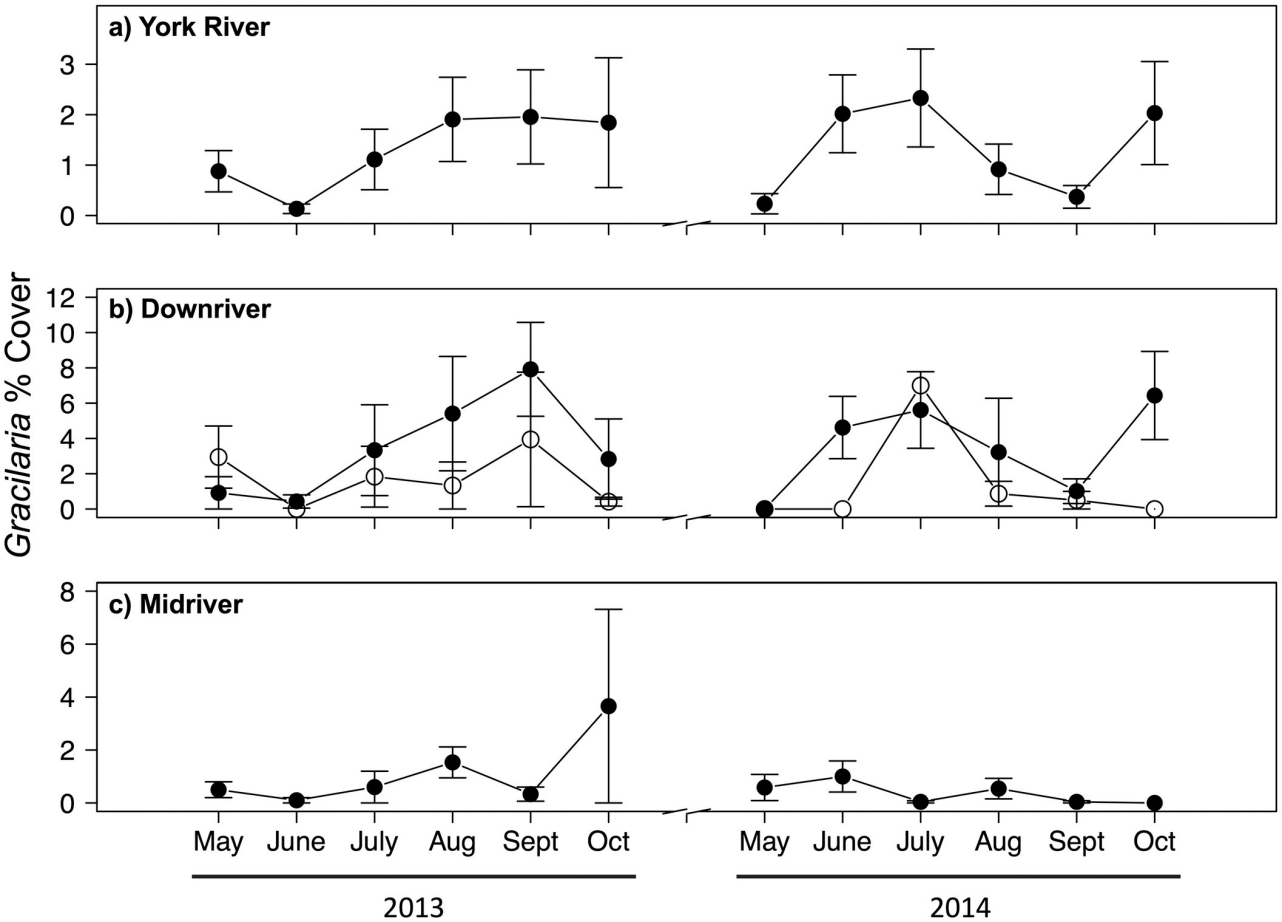
Mean *Gracilaria vermiculophylla* percent cover during 2013 and 2014 over: (a) the entire York River; (b) the downriver region, where base habitats are seagrass (filled) and unvegetated (open); and (c) midriver region. *Gracilaria vermiculophylla* was not observed upriver. Vertical bars represent 1 standard error of the mean. Note differing scales on y-axes.

**Fig 6:**
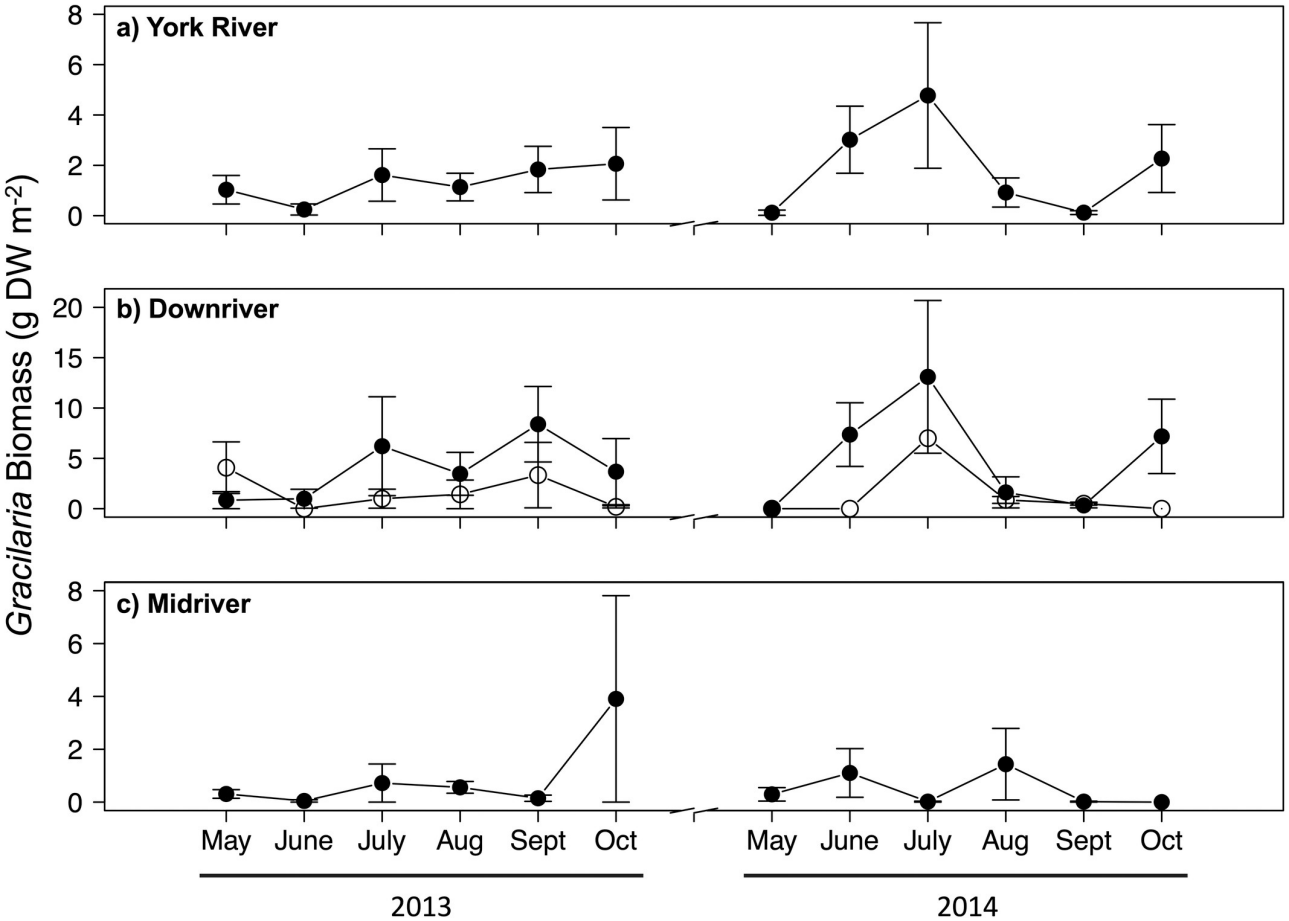
Mean *Gracilaria vermiculophylla* biomass (g dry weight [DW] m^−2^) during 2013 and 2014 over: (a) the entire York River; (b) the downriver region, where base habitats are seagrass (filled) and unvegetated (open); and (c) midriver region. *Gracilaria vermiculophylla* was not observed upriver. Vertical bars represent 1 standard error of the mean. Note differing scales on y-axes.

The patterns of *Gracilaria* percent cover (Fig 5) and biomass (Fig 6) followed those of *Gracilaria* presence (Fig 3). As with presence, *Gracilaria* percent cover and biomass were higher downriver and in seagrass beds (Figs 5 and 6), although their magnitudes varied between years as did presence. For instance, in 2013 during the July through September recruitment period, *Gracilaria* percent cover averaged about 6% in downriver seagrass, 3% in downriver unvegetated bottom, and 1% in midriver unvegetated bottom. These values of percent cover may seem low, but the areal extent of habitats where *Gracilaria* occurred was on the scale of 100s of hectares.

As with presence and percent cover, algal biomass was greater in 2014 than 2013 (Fig 6a). In 2013, there was a plateau of relatively higher biomass from July–October, whereas in 2014 biomass was highest from June–July. Within regions, algal biomass was greater downriver in 2014 than 2013 (Fig 6b), likely because salinity downriver was higher in 2014 than in 2013 (Table 1). Algal biomass was also greater midriver in 2013 than 2014 (Fig 6c). Downriver, biomass was greatest in July 2014 (11.9 g DW m^−2^), and, while algal biomass was relatively stable in 2013, it was variable in 2014. Midriver, algal biomass was greatest in October 2013 (3.9 g DW m^−2^), and patterns of biomass were similar between years.

As for presence, the best-fitting models for percent cover (Table 5) and biomass (Table 6) only included habitat. *Gracilaria* percent cover (3.52%) in downriver seagrass was twice that in downriver sand (1.67%) and 3.4 × higher than in midriver sand (0.80%) (Fig 4B). *Gracilaria* percent cover in downriver sand was twice that in midriver sand (Fig 4B). On average, *Gracilaria* biomass (4.7 g DW m^−2^) was over 3 × higher in downriver seagrass than in downriver sand (1.5 g DW m^−2^) and nearly 6 × higher than in midriver sand (0.8 g DW m^−2^) (Fig 4C). *Gracilaria* biomass in downriver sand was almost twice as high as in midriver sand (Fig 4C).

**Table 5:** Summary of GLM for model g_7_ predicting percent cover, including parameter estimates (log-transformed), standard errors, *z* values and *p* values. The intercept represents downriver seagrass. Model *g*_7_ explained 12.9% of the null deviance.

Coefficients	Estimate	SE	*z*	*p*
Intercept	0.54	0.25	2.16	0.03
Downriver sand	-0.84	0.41	-2.08	0.04
Midriver sand	12.69	0.34	-4.97	≪ 0.01

**Table 6:** Summary of GLM for model g_7_ predicting plant biomass, including parameter estimates, standard errors, *z* values and *p* values. The intercept represents downriver seagrass. Model *g*_7_ explained 16.5% of the null deviance.

Coefficients	Estimate	SE	*t*	*p*
Intercept	1.55	0.06	27.76	≪ 0.01
Downriver sand	-1.15	0.14	-8.17	≪ 0.01
Midriver sand	-1.82	0.13	-14.53	≪ 0.01

### Juvenile crab density and size

Of the 12 candidate models for density (Table 2), model g_2_ had the highest w_*i*_ = 0.34, although g_5_ deserved consideration because its w_*i*_ = 0.27 exceeded 0.1 (Table 3). Model g_2_ included the additive effects of plant % cover, habitat type and month; plant % cover and month were significant, but habitat type (*Gracilaria* or seagrass) was not (Table 7). Model g_5_, which added plant biomass as an extra parameter, was eliminated because it had a lower w_*i*_ than g_2_, and did not improve the fit even with one extra parameter (LR test, *p* = 0.17). The global model g_11_ also did not improve the fit over g_2_ (LR test, *p* = 0.85). In contrast, model g_2_ produced a significantly better fit than the null model g_12_ (LR test, *p* ≪ 0.001).

**Table 7:** Summary of GLM for model g_2_ predicting crab density, including parameter estimates, standard errors (SE), *t* values and *p* values. The intercept represents August *Gracilaria*. Model *g*_2_ explained 52.2% of the null deviance.

Coefficients	Estimate	SE	*t*	*p*
Intercept	1.52	0.45	3.38	0.002
Plant % cover	0.017	0.006	2.64	0.012
Month = June	-1.69	0.26	-6.43	<0.001
Habitat = seagrass	0.103	0.252	0.41	0.686

**Fig 7:**
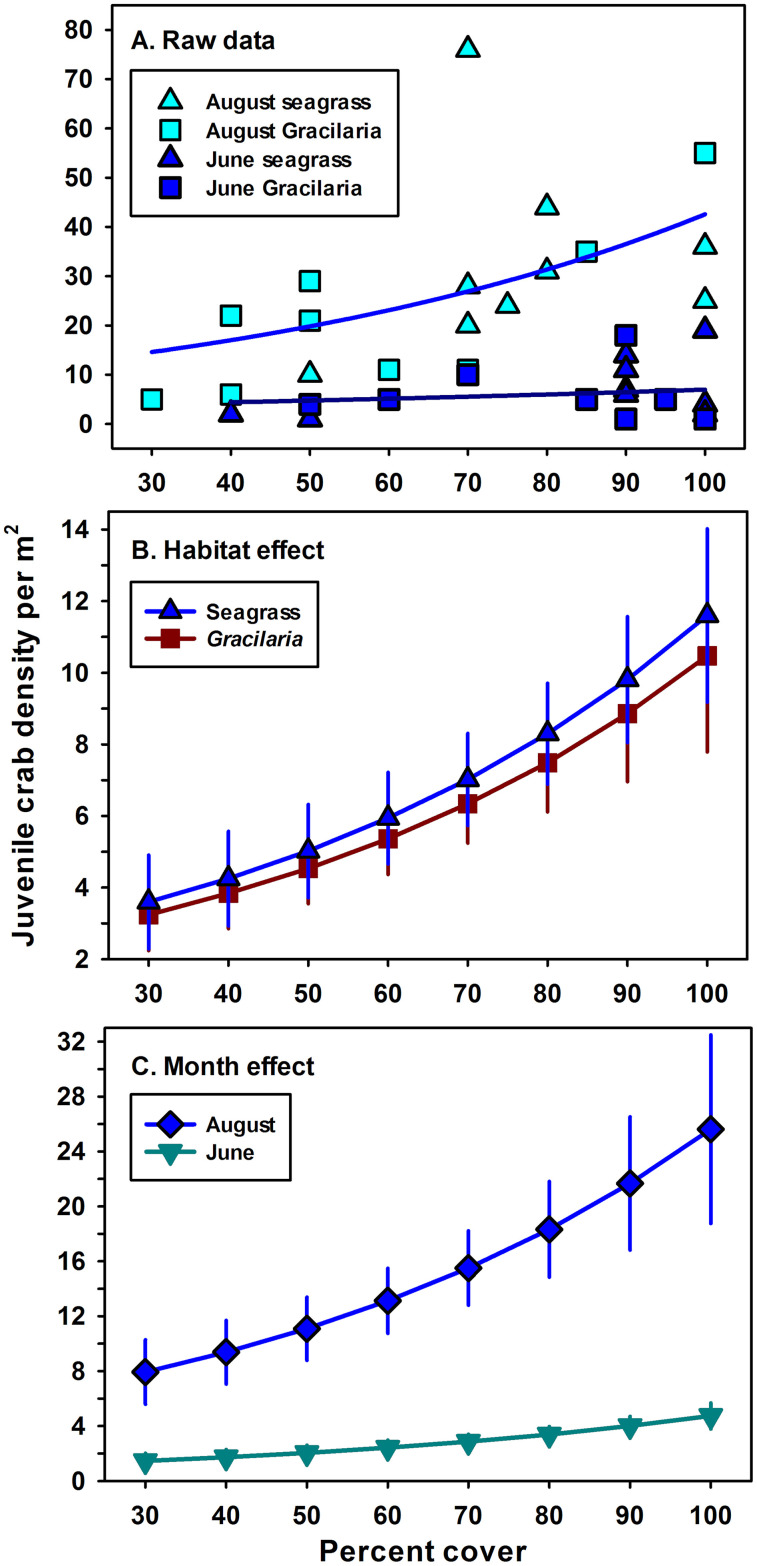
Juvenile crab density as a function of plant % cover, month, and habitat type using parameter estimates from model *g*_2_ (Table 3). (A) Unadjusted crab densities with separate exponential fits to the raw data for August and June. Note that the curves do not account for the effect of habitat type. (B) Crab densities as a function of habitat type after accounting for the effect of month. (C) Crab densities as a function of month after accounting for the effect of habitat type. Error bars = ± 1 SE.

Juvenile crab density in 2013 was an exponentially increasing function of plant percent cover (Fig 7); at 100% cover, crab density was nearly four-fold higher than crab density at 30% cover (Fig 7B). Juvenile crab densities were also significantly greater, over four-fold, in August than in June (Fig 7C) due to the influx of the 2013 cohort of new Age-0 recruits in July and August. Although juvenile crab densities were slightly higher in seagrass than in *Gracilaria* (Fig 7A and 7B), they did not differ significantly (Table 7).

**Fig 8:**
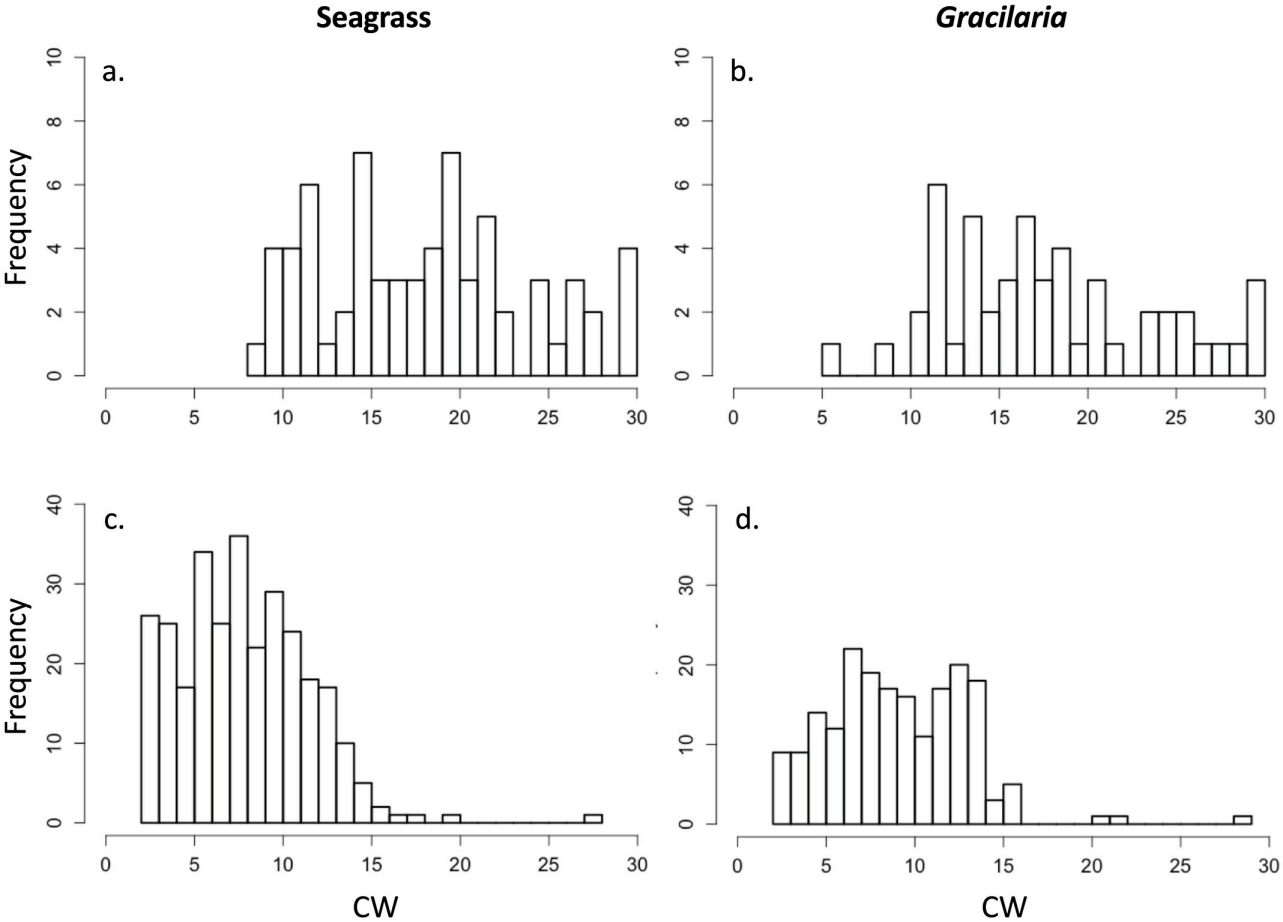
Juvenile blue crab size frequencies in seagrass and *Gracilaria vermiculophylla* in June (a and b, respectively) and August (c and d, respectively). Note differing scales on y-axes.

In June 2013, juvenile blue crab size did not differ significantly between *Gracilaria* and seagrass (Table 8). These crabs represented the 2012 cohort that recruited from July through November 2012 and which were captured in June 2013. In August, crab size was significantly smaller by 13% in seagrass (7.9 vs. 9.1 mm CW, Table 8). In particular, juveniles from 2.5 to 4.0 mm CW (J1 and J2 benthic instar crabs) were 2.8 times more abundant in seagrass than in *Gracilaria* (Fig 8).

**Table 8:** Mean juvenile crab size (<30 mm CW) in seagrass and *Gracilaria vermiculophylla* in June and August 2013, with standard error and 95% confidence interval.

Habitat	June				August			
	Mean	SE	95% CI	n	Mean	SE	95% CI	n
**Seagrass**	18.0	0.7	(16.6, 19.4)	10	7.9	0.2	(7.5, 8.3)	9
***Gracilaria***	17.8	0.9	(16.1, 19.5)	9	9.1	0.3	(8.6, 9.6)	9

## Discussion

### *Gracilaria* distribution and biomass patterns

Unlike previous studies [38, 72], salinity was associated with the distribution of *Gracilaria vermiculophylla*. Greatest abundance occurred at the higher salinities characterizing downriver and midriver regions of the York River. While the upriver region falls within the salinity tolerance and growth range of *G. vermiculophylla* (5–60) and other species of *Gracilaria* [73–77], the alga has not yet colonized this region. In Sweden, *Gracilaria* expanded its range by about 150 km over two years [48], indicating that it is an efficient colonizer, so it is possible that the alga has not colonized the upriver region due to physical constraints like currents or other environmental factors. Sexual reproduction and spore release is uncommon in *Gracilaria*’s non-native range [78]; therefore, the alga would likely spread farther upriver where the subtidal habitat is predominantly unvegetated via fragmentation or entanglement in fishing gear or boat anchors.

*Gracilaria* was also more abundant where seagrass occurred. Seagrasses may create favorable conditions for the alga or have similar environmental requirements as *Gracilaria*. Because seagrasses are only present in the downriver region of the York River where salinity is highest, the presence of seagrass covaries with high salinity and, thus, may partially explain why salinity was correlated with *Gracilaria*. However, *Gracilaria* presence and biomass were also greater downriver than midriver at unvegetated sites, indicating that salinity or some other environmental factor associated with salinity, may play a major role in the distribution of the alga. The increased presence, biomass, and cover of the alga in areas with seagrass was likely due to the propensity of the drifting algal fragments to become entangled in seagrass. In unvegetated areas where there is little structure to encourage entanglement of drifting thalli or spore settlement, the alga has a patchy distribution and large floating mats are rare. Typically, the alga is found as drifting thalli or, more commonly, fragments may be incorporated into polychaete tubes [10, 12].

The presence, biomass, and percent cover of *Gracilaria* varied over time, which is consistent with a fast-growing alga that easily fragments. It is likely that differences in biomass between years were due to differences in environmental variables like storm activity, temperature, salinity, and nutrient inputs. For instance, intensified storm activity may increase the likelihood of algal fragmentation, and winds may push these fragments into very shallow areas of low flow. Fragments and mats may also be advected into deeper water and, due to the negative buoyancy of the alga, be removed from the system. In the Baltic Sea, *Gracilaria* biomass increased 3-fold over 2 y, while algal biomass increased by a factor of 18.5 in field experiments, indicating a potentially large sink for algal biomass in deep water (>2 m depth) [76].

### Interactions with native seagrasses

While *Gracilaria* presence and biomass covaried with seagrass presence, algal biomass in this study was moderate and likely below the level at which negative impacts on seagrass occur [79]. Negative effects of *Gracilaria* on seagrass may be exacerbated due to climate change and other anthropogenic impacts. Increased sea surface temperatures and eutrophication will likely cause reduced growth and increased mortality of seagrasses with concurrent increases in growth rates of algal species [22, 80, 81]. In Chesapeake Bay, eelgrass *Zostera marina* is already experiencing periodic mass mortality events due to above average summer water temperatures combined with other environmental stressors like increased turbidity [82]. Thus, *Gracilaria* may increasingly impact seagrasses due to their interaction. For instance, *Gracilaria* tends to exacerbate the negative effects of elevated temperature regimes (26–30°C) on *Z. marina*, which experiences decreased growth and increased mortality [83, 84]. In contrast, growth of *Gracilaria* is positive over a range of temperatures (5–30°C) with maximum growth from 15–25°C [73, 74, 77]; decreased growth and increased mortality only occur at temperatures exceeding 32.5°C [74, 77].

Unlike eelgrass, widgeon grass *Ruppia maritima* is more tolerant of higher water temperatures, such that growth increases with temperature from 8 to 30°C [85], suggesting that this species could potentially also compensate for eelgrass loss [86]. On the west coast of North America, widgeon grass replaced eelgrass after a period of increased water temperature during an El Niño event [87]. However, *R. maritima* is limited to shallower areas than *Z. marina* and is more susceptible to physical disturbances like waves and storms [88], suggesting that it will not replace eelgrass in all areas of Chesapeake Bay. Additionally, widgeon grass has not recolonized areas midriver where seagrasses have been lost [64, 86], and where *Gracilaria* has become the dominant subtidal vegetation. Differences in optimal growth conditions between *Z. marina* and both *R. maritima* and *G. vermiculophylla* indicate that the shallow subtidal structural habitat of lower Chesapeake Bay may shift from one dominated by eelgrass to one dominated by algae and widgeon grass as sea surface temperatures increase with climate change.

### Implications for juvenile blue crabs

Small juvenile blue crabs use *Gracilaria* as a primary and early secondary nursery habitat [33]. *Gracilaria* is present in Chesapeake Bay shallow-water habitats where megalopae settle and which juveniles colonize in late summer and fall [89]. The alga is also present in late spring when crabs that recruited in late fall have overwintered and are still of a size (<30 mm CW) that uses structured nursery habitats. However, variable algal biomass indicates that *Gracilaria* may not represent a continuously stable nursery habitat, so juvenile blue crabs may use it opportunistically when it is available, especially downriver and midriver where juvenile densities can be high [27] and density-dependent dispersal from nursery habitats is more probable [58, 90].

Densities of juvenile blue crabs were similar in *Gracilaria* and seagrass in June and August, supporting the hypothesis that the alga is used by young juvenile blue crabs as nursery habitat. There were, however, almost 3 × as many small juvenile crabs (J1 and J2) in seagrass as compared to algal habitat in August, shortly after recruitment, suggesting that megalopae preferentially settle in seagrass rather than in the alga. Juvenile crab densities reported here are similar to those observed previously in both seagrass and *Gracilaria* [33]. While our study and the previous study were conducted in the York River, the spatial distribution of vegetated samples was broad in our study, thereby validating the conclusion of widespread distribution of *Gracilaria* in the midriver and downriver sections of the York River. In addition, juvenile crab density positively correlated with percent cover of both *Gracilaria* and seagrass, similar to the results of a previous study in Chesapeake Bay [91].

**Fig 9:**
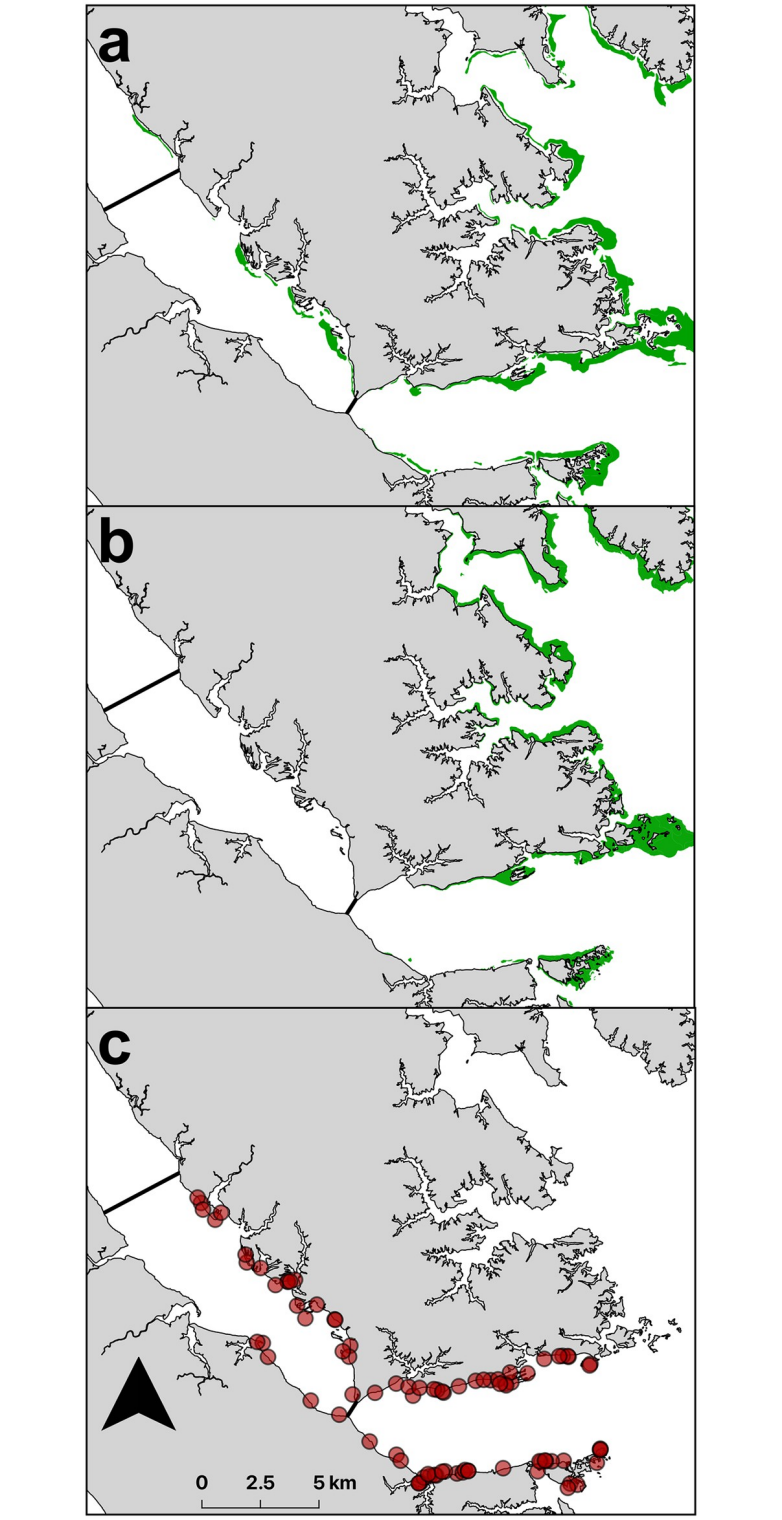
Locations within the York River of (a) seagrass cover (green) in 1971 [92], (b) seagrass cover (green) in 2014 [66], and (c) sites where *Gracilaria vermiculophylla* was present in both 2013 and 2014.

*Gracilaria vermiculophylla* is providing the only vegetated subtidal nursery habitat midriver where seagrass has been extirpated (Fig 9). This change from unvegetated to vegetated substrate adds structural complexity to these shallow subtidal areas and may represent an important emerging nursery habitat for juvenile crabs in these lower salinity areas where megalopal settlement and juvenile secondary dispersal rates can be high [32]. The estimates of *Gracilaria* percent cover may seem low, but the areal extent of habitats where *Gracilaria* occurred was on the scale of 100s of hectares. For example, a typical muddy cove system, where *Gracilaria* occurred in the York River, encompasses approximately 40 ha, such that a downriver cove with 6% *Gracilaria* percent cover would be equivalent to one of the four large 2.4-ha seagrass beds near the mouth of the York River. Using crab densities quantified in this study, both a cove system and a single seagrass bed could house about 200,000 juvenile crabs. Consequently, the numerous midriver and downriver cove systems in the York River (Fig 9) could support millions of young juvenile blue crabs and thereby compensate for the loss of seagrass in the river and in other areas of Chesapeake Bay.

## Supporting information

S1 Fig Relationship between *Gracilaria vermiculophylla* volume and biomass with linear regression. (TIF)
