# Supplementary figures and images for: Non-native red alga Gracilaria vermiculophylla compensates for seagrass loss as blue crab nursery habitat in the emerging Chesapeake Bay ecosystem
Source: PLoS One. 2022 May 31;17(5):e0267880. doi: 10.1371/journal.pone.0267880 (PMC9154113; doi:10.1371/journal.pone.0267880)

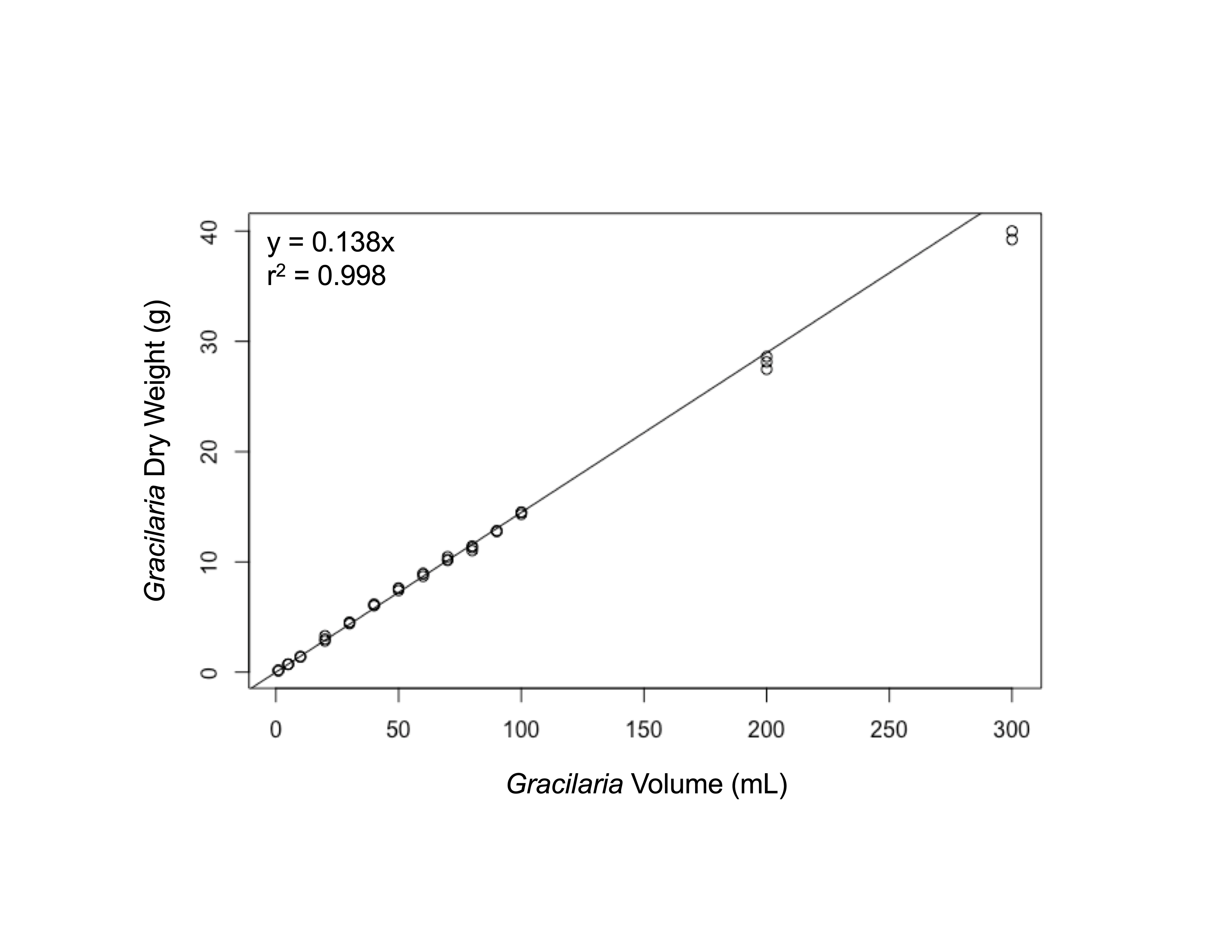

Supplement: S1 Fig — (TIF) [file pone.0267880.s001.tif]
